# Electrocardiogram Performance in the Diagnosis of Left Ventricular Hypertrophy in Hypertensive Patients With Left Bundle Branch Block

**DOI:** 10.5935/abc.20160187

**Published:** 2017-01

**Authors:** Paula Freitas Martins Burgos, Bráulio Luna Filho, Francisco de Assis Costa, Maria Teresa Nogueira Bombig, Dilma de Souza, Henrique Tria Bianco, Japy Angelini Oliveira Filho, Maria Cristina de Oliveira Izar, Francisco Antonio Helfenstein Fonseca, Rui Póvoa

**Affiliations:** 1 Universidade Federal de São Paulo (UNIFESP) - Brazil; 2 Universidade Federal do Pará (UFPA), Belém, PA - Brazil

**Keywords:** Hypertension, Electrocardiography / methods, Hypertrophy, Left Ventricular / diagnosis, Bundle-Branch Block

## Abstract

**Background:**

Left ventricular hypertrophy (LVH) is an important risk factor for cardiovascular events, and its detection usually begins with an electrocardiogram (ECG).

**Objective:**

To evaluate the impact of complete left bundle branch block (CLBBB) in hypertensive patients in the diagnostic performance of LVH by ECG.

**Methods:**

A total of 2,240 hypertensive patients were studied. All of them were submitted to an ECG and an echocardiogram (ECHO). We evaluated the most frequently used electrocardiographic criteria for LVH diagnosis: Cornell voltage, Cornell voltage product, Sokolow-Lyon voltage, Sokolow-Lyon product, RaVL, RaVL+SV_3_, RV_6_/RV_5_ ratio, strain pattern, left atrial enlargement, and QT interval. LVH identification pattern was the left ventricular mass index (LVMI) obtained by ECHO in all participants.

**Results:**

Mean age was 11.3 years ± 58.7 years, 684 (30.5%) were male and 1,556 (69.5%) were female. In patients without CLBBB, ECG sensitivity to the presence of LVH varied between 7.6 and 40.9%, and specificity varied between 70.2% and 99.2%. In participants with CLBBB, sensitivity to LVH varied between 11.9 and 95.2%, and specificity between 6.6 and 96.6%. Among the criteria with the best performance for LVH with CLBBB, Sokolow-Lyon, for a voltage of ≥ 3,0mV, stood out with a sensitivity of 22.2% (CI 95% 15.8 - 30.8) and specificity of 88.3% (CI 95% 77.8 - 94.2).

**Conclusion:**

In hypertensive patients with CLBBB, the most often used criteria for the detection of LVH with ECG showed significant decrease in performance with regards to sensitivity and specificity. In this scenario, Sokolow-Lyon criteria with voltage ≥3,0mV presented the best performance.

## Introduction

Left ventricular hypertrophy (LVH) diagnosis by electrocardiogram (ECG) in hypertensive patients involves clinical and prognostic decisions. Pioneering studies by Framingham have shown that alterations in QRS voltage and ventricular repolarization are important determining factors for cardiovascular events.^1,2^

Despite its relatively low sensitivity, ECG makes up for this limitation with high specificity in the identification of LVH. Moreover, it is a widely used method that is easily accessible and low cost. However, several situations may negatively alter ECG performance in LVH diagnosis, among which is the presence of complete left bundle branch block (CLBBB).^3^ Because it interferes in the measurement of its criteria or parameters, alterations promoted by CLBBB in ECG tracings are described as restrictive for the electrocardiographic diagnosis of LVH.

The objective of this study was to evaluate CLBBB influence in the sensitivity and specificity of the main electrocardiographic criteria used in LVH diagnosis in patients with systemic arterial hypertension (SAH).

## Methods

We analyzed ECG tracings in 12-lead of 2,240 hypertensive patients in outpatient care. Patients with valvular diseases, known coronary artery disease, previous myocardial infarction, Chagas disease, rhythm disturbances, right bundle branch block, use of digitalis compounds, ventricular pre-excitation, or inadequate technical quality of the echocardiogram were excluded from the present analysis.

### Electrocardiogram

All participants were submitted to a 12-lead ECG at rest, recording at a speed of 25 mm/s and standardized calibration for 10 mm/cm (equipment - Dixtal^®^ EP3, Brazil). For the precise analysis of the tracing, we used a magnifying glass that allowed a fivefold enlargement in its contact face. In all tracings (analyzed by the same observer), a certified cardiologist with experience in ECG reading was brought in. We estimated the axis and duration of the QRS complex; R wave amplitude in aV_L_, V_5_ and V_6_ leads; S wave amplitude in V_1_, V_2_ and V_3_; and the strain pattern in V_5_ e V_6_. We separately analyzed 14 electrocardiographic criteria for LVH:

- Cornell voltage criteria: RaV_L_ + SV_3_ ≥20 mm for women and ≥28 mm for men.^4^
- Cornell criteria duration: (RaV_L_ + SV_3_) x QRS duration - for women, add 8 mm, ≥2440 mm.ms.^5^
- Sokolow-Lyon voltage criteria: SV_1_ + RV_5_ or V_6_ ≥30 mm and ≥35 mm.^6^
- Sokolow-Lyon product criteria: (SV_1_ + RV_5_ or V_6_) x QRS duration ≥3710 mm.ms.^7^
- Gubner-Ungerleider score: RD1+SV3 >25 mm.^8^
- R wave of aV_L_ ≥ 11 mm.^9^
- RaVL product: RaVL x duration QRS ≥1030 mm.ms.^7^
- RaVL +SV_3_ >16 mm in men and >14mm in women.^10^
- RV_6_/RV_5_ ratio >1.^11^
- (Biggest R wave + biggest S wave) x (QRS duration): >28 mm.ms.^12^
- Presence of the strain pattern: defined as the convex depression of the ST segment with asymmetrical inversion of the T wave opposed to QRS complex in V_5_ or V_6_ leads.^13^
- Left atrial enlargement: duration ≥120 ms; P wave alteration at D2 with slurrying in the apex or Morris signal in V1; terminal component with duration and amplitude ≥ 0,04 mm.s).^14^

### Other analyzed electrocardiographic variables

- QT interval: measured in ms, from the beginning of the Q wave to the end of the T wave (corrected through Bazett's formula: QTc = QT/RR^1/2^; normal values from 350 to 440 ms).^15^
- CLBBB was identified when: duration off the QRS ≥120ms; absence of "q" wave in D1, aVL, V5 and V6; widened R waves with slots and/or medium-terminal slurrying in D1, aVL, V5 and V6; "r" wave with slow growth of V1 to V3 with possible occurrence of QS; widened S waves with thickening and/or slots in V1 and V2; intrinsicoid deflection in V5 and V6 ≥0,05 s, electrical axis between -30º e + 60º; ST depression and asymmetrical T wave in opposition to medium-terminal delay.^16^

### Transthoracic echocardiogram

The exams were performed with the device ATL^®^ 1500, USA, with 2.0 and 3.5 MHz transducers. All measurements were obtained by the same observer who was unaware of participants' clinical characteristics, and according to the recommendations of the European Association of Echocardiography.^17^ Images were obtained with the participant in left lateral decubitus from the left parasternal region between the fourth and fifth intercostal space, proceeding with the habitual sections for the complete study in M and two-dimensional modes, simultaneously with the recording of the ECG. According to the recommendations of the Penn Convention, the following measurements were performed: left ventricle size (LV) in systole and diastole; interventricular septum thickness in diastole (IVSD) and end diastolic left ventricular posterior wall thickness (LVPWd); LV end-diastolic diameter (LVDd); end systolic and diastolic volumes, and percentage of diastolic shortening and ejection fraction by the cube method. LV mass was calculated by the formula: LV mass = 0.8 X {1.04 [(IVSD + LVDd + LVPWd)^3^ - (LVDd)^3^]} + 0.6 g.^17^ LV mass was indexed for body surface to adjust differences in heart size depending on the patient size. Body surface was calculated by the formula BS = (W - 60) X 0.01 + H, where: BS is the body surface in m^2^, W is the weight in kg, and H is the height in meters.^18^ Enlargement of the LV mass was considered when the mass index was ≥96 g/m^2^ for women and ≥116 g/m^2^ for men.

### Statistical analysis

Continuous variables were expressed in mean and standard deviation. Categorical variables were expressed in percentages. We used Pearson's linear correlation coefficient to determine the association between LVMI and the numerous electrocardiographic criteria. To analyze the performance of LVH electrocardiographic criteria, we used the values obtained for sensitivity and specificity with the respective confidence intervals of 95%. In the evaluation of statistical differences between LVH electrographic criteria in patients with and without CLBBB, we used McNemar's paired test. A reproducibility study of ECG tracings was performed by three observers who interpreted 100 tracings randomly taken from the sample. To that end, we analyzed the amplitude of R and S waves and the duration of the QRS complex, and the Kappa test was used.^19^ To verify statistical significance, in all comparisons, we considered confidence intervals of 95% and p < 0.05. All analyses were executed with the software SPSS (version 17.0, SPSS Inc., Chicago, IL, USA).

## Results

Of the 2,240 studied participants, 684 were male (30.5%), and 1,556 were female (69.5%), with a mean age of 11.3 ± 58.7 years. Of these, 2,054 (91.7%) constituted the group of patients without CLBBB, and 186 (8.3%) formed the group with CLBBB. In the group without CLBBB, 46.8% had LVH whereas in the group with CLBBB, 67.7% had LVH, as shown in Table 1. In this series, we had 11.8% (22/186) of the patients with CLBBB with left anterior divisional block.

**Table 1:** Characteristics of the sample according to the presence or absence or CLBBB

No CLBBB (n=2054)		With CLBBB (n=186)	
Age	Male / Female	Age	Male / Female
11.4±58.3	610 (29.7%) / 1444 (70.3%)	8.5±63.4	74 (39.8%) / 112 (60.2%)

Data expressed as mean and standard deviation and n (%). CLBBB: complete left bundle branch block.

According to Pearson's correlation, in both groups there was a significant association between LVMI and the electrocardiographic variables for most LVH criteria (Table 2). However, the correlations between the several criteria and LVMI showed a moderate or weak correlation, suggesting that these criteria are not fully able to explain the presence of LVH, regardless of CLBBB in the electrocardiographic tracing. We did not perform correlations between LVMI with enlargement of the left atrium and the strain pattern considering these are qualitative variables.

**Table 2:** Pearson correlation between LVMI and the analyzed electrocardiographic criteria

Variable	Without CLBBB (n=2054)	With CLBBB (n=186)
Cornell voltage	0.400*	0.306*
Cornell duration	0.456*	0.392*
Sokolow-Lyon voltage	0.404*	0.124
R in aVL	0.300*	0.141
QTc	0.085*	0.210*
Gubner-Ungerleider	0.536*	0.305*
(Rmax+Smax) x dur QRS	0.546*	0.383*

* p< 0.05; LVMI: left ventricular mass index; CLBBB: complete left bundle branch block..

In relation to the electrocardiographic criteria for LVH, patients with CLBBB presented significant alterations with expressive decrease in values. Sokolow-Lyon voltage criteria (≥3.0 mV e ≥3.5 mV), R wave amplitude in aVL, and enlargement of the left atrium had the lowest reductions in specificity. Interestingly, this happened with an insignificant alteration in sensitivity (Tables 3 and 4). In the criteria in which there were substantial increases of sensitivity indices, such as Cornell voltage and Cornell voltage product, these increases were concomitant with the expressive loss of specificity, which hinders the application of these criteria in the scenario of ECG with the presence of LBBB.

**Table 3:** Sensitivity of electrocardiographic variables for LVH in patients with and without CLBBB

Criteria	Without CLBBB (n=2054)	With CLBBB (n=186)	p
	Sensitivity (CI^95%^)	Sensitivity (CI^95%^)	
Sokolow-Lyon voltage ≥35 mm	12.5 (10.6-14.8)	12.7 (7.90-19.6)	ns
Sokolow-Lyon voltage ≥30 mm	21.0 (18.5-23.6)	22.2 (15.8-30.8)	ns
Sokolow-Lyon duration ≥3710 mm.ms	7.6 (6.1-9.5)	46.8 (38.3-55.5)	*
Cornell Voltage ≥ 28 mm (m). ≥20 (f)	9.3 (7.6-11.3)	78.5 (67.6-86.5)	*
Cornell Voltage duration 2440 mm.ms	17.4 (15.2-19.9)	86.5 (79.4-91.4)	*
Gubner-Ungerleider ≥ 25 mm	33.2 (30.3-36.3)	59.5 (50.7-67.6)	*
RaVL ≥ 11 mm	10.0 (8.3-12.1)	11.9 (7.3-18.7)	ns
RaVL duration >103 mm.ms	8.9 (7.3-10.9)	46.0 (37.5-54.7)	*
RaVL+SV3 >16 mm (m). 4 mm (f)	40.9 (37.8-44.0)	88.1 (81.4-92.7)	*
QTc ≥ 440 ms	35.4 (32.4-38.5)	80.9 (73.2-86.8)	*
V6/V5 >1	12.4 (10.5-14.7)	72.3 (72.3-86.1)	*
(Rm+Sm) product ≥28 mm.ms	30.8 (28.0-33.8)	95.2 (90.0-97.8)	*
Enlarged left atrium	38.1 (35.1- 41.2)	32.5 (24.9-41.1)	ns
Strain pattern	16.6 (14.4-19.1)	51.5 (42.9-60.1)	*

* increase in sensitivity with p value < 0.05; CI 95%: confidence interval; ns: non-significant; m: male; f: female. LVH: Left ventricular hypertrophy;CLBBB: complete left bundle branch block.

**Table 4:** Specificity of electrocardiographic variables for LVH in patients with and without CLBBB

Criteria	Without CLBBB (n=2054)	With CLBBB (n=186)	p
	Specificity (CI^95%^)	Specificity (CI^95%^)	
Sokolow-Lyon voltage ≥35 mm	97.6 (96.5-98.3)	96.6 (88.6-99.0)	ns
Sokolow-Lyon voltage ≥30 mm	92.4 (90.7-93.9)	88.3 (77.8-94.2)	ns
Sokolow-Lyon product ≥3710 mm.ms	99.1 (98.4-99.5)	70.0 (57.4-80.1)	*
Cornell Voltage	99.2 (98.5-99.6)	38.2 (29.8-47.3)	*
Cornel Voltage product ≥28 mm (m). ≥20 mm (f)	96.7 (95.5-97.6)	20.3 (12.0-32.2)	*
Gubner-Ungerleider ≥ 25 mV	91.1 (89.2-92.6)	61.6 (49.0-72.9)	*
RaVL ≥ 11 mm	97.0 (95.8-97.2)	96.6 (88.6-99.0)	ns
RaVL.durQRS >103	98.5 (97.6-99.1)	71.6 (59.2-81.4)	*
RaVL+SV3 >16 mm (m). 14 mm (f)	84.2 (81.9-86.2)	18.3 (10.5-29.9)	*
QTc ≥ 440 ms	70.2 (67.4-72.8)	25.0 (15.7-37.2)	*
V6> V5	90.9 (89.0-92.5)	18.3 (10.5-29.9)	*
(Rm+Sm) product ≥28 mm.ms	90.4 (88.5-92.0)	6.6 (2.6 -15.9)	*
Enlarged left atrium	77.8 (75.2-80.2)	75.0 (62.7-84.2)	ns
Strain Pattern	97.7 (96.6-98.4)	50.0 (37.3-62.1)	*

* decrease in specificity with p value < 0.05; CI 95%: confidence interval; ns: non-significant; m: male; f: female. LVH: Left ventricular hypertrophy;CLBBB: complete left bundle branch block.

With regards to the reproducibility study, the level of agreement among the three observers varied between 0.82 and 0.98, which are considered excellent numbers. The first figure corresponds to the duration of the QRS complex, and the last one to the amplitude of R and S waves, respectively.

## Discussion

The presence of LVH is a consistent predictor of high cardiovascular risk, regardless of other comorbidities. In clinical and epidemiological studies, there is a clear relation between LVH and adverse cardiovascular events. Hence the importance of early detection, if possible, through low-cost, easily accessible diagnostic methods. Unquestionably, ECG is one of the most frequently used methods in the detection of LVH, be it for its low operational cost or wide availability. It is often an initial instrument in the identification of several cardiologic manifestations. In the scenario of LVH secondary to SAH, it is inarguably the most cost-effective exam. It is known, however, that several factors interfere in the diagnostic precision of LVH, more specifically the presence of conduction disturbances, especially CLBBB, which notoriously imposes limitations in LVH diagnosis.^20-22^

In the last few decades, ECHO has become the reference exam in the evaluation of LV mass and function. In this context, it is used not only to confirm LVH, but also other pathological manifestations. As opposed to ECG, ECHO found the limitation in LVH identification, and provided earlier diagnosis and more aggressive approaches to associated diseases, such as SAH. However, despite its relatively low sensitivity, ECG is still the most widely used exam to detect LVH in hipertensive patients. This is because it is an easily performed test that shows excellent inter/ intraobserver reproducibility. Conversely, besides having a much higher operational cost, ECHO is extremely dependent not only on the quality of the device, but also on the observer interpreting the images.

Since CLBBB interferes in several electrocardiographic parameter employed in LVH diagnosis, in this study we evaluated the main criteria used by the ECG in this situation.^23^ Considering LV mass calculation presumes the heart to be in normal, ellipsoid shape, patients with dilated hearts were excluded. To increase homogeneity in the analysis of sample members, we used LVMI to compare individuals with different body compositions and, thus, obtain values that would better identify groups at high risk for cardiovascular events.^24-26^

LVMI association with LVH electrocardiographic criteria showed moderate or weak correlation in patients with and without CLBBB. However, in the group with CLBBB, even though Sokolow-Lyon voltage and RaVL criteria did not show statistically significant correlation with LVMI, they presented the best diagnostic performances.

In patients with CLBBB, sensitivity varied between 12.7% and 95.2%, and specificity between 6.6 and 96.6%. The electrocardiographic criteria that predominantly used QRS complex voltage presented an increase in sensitivity, but at the cost of a great reduction in specificity. We observed that the criteria that obtained the highest sensitivity increases, such as Cornell criteria, RaVL duration, RaVL+SV_3_, also had the highest statistically significant reduction in specificity. Exceptions included only Sokolow-Lyon voltage and RaVL, which had discreet, non-significant reductions in specificity.

Generally speaking, there was a reduction in specificity, with mild or strong intensity, in all the criteria. However, among the criteria that showed the best performance in detecting LVH in the presence of CLBBB, Sokolow-Lyon for a voltage of ≥3,0mV with a sensitivity of 22.2% (CI 95% 15.8 - 30.8) and specificity of 88.3% (IC 95% 77.8 - 94.2) stood out. We would point out that these values have no statistical significance. It is known that sensitivity and specificity data are related to the prevalence of the phenomenon in the evaluated sample. It is also known that hypertensive patients with CLBBB are usually older and have had the disease for longer. This explains why, in the present study, the group of patients with CLBBB presented a prevalence of 67.7%. Conversely, the group without CLBBB have a lower prevalence (46.8%).

The reasons for the different performances of the several electrocardiographic criteria are not clear. However, they are related to the specificity of parameters that compose each criterion, with the limitations of each method, which essentially stem from the electrical activity of the cardiac muscle and are, deductively, correlated to the three-dimensional anatomic alteration. Moreover, besides the specific limitations of each criteria in particular, there are also individual characteristics of the studied sample.

## Conclusion

CLBBB modifies ECG sensitivity and specificity in the detection of LVH. However, the best diagnostic performance of the ECG, in the presence of CLBBB, occurred with Sokolow-Lyon voltage and RaVL criteria. The other electrocardiographic criteria presented expressive losses in specificity, rendering them less indicated in the presence of this conduction disturbance. Considering this is a study performed in a relatively young, hypertensive population in outpatient care, caution is recommended when transferring these results onto a group of older patients with more advanced hypertensive diseases.
